# Manually prepared lamellae for Descemet stripping endothelial keratoplasty (Pachy-DSEK): comparison of four dissection depths

**DOI:** 10.5935/0004-2749.2022-0076

**Published:** 2024-12-18

**Authors:** Pedro Bertino, Renata Soares Magalhães, Carlos Jose de Souza Junior, Lucio de Vieira Leite Maranhão, Tatiana Moura Bastos Prazeres

**Affiliations:** 1 Hospital de Olhos INOB, Brasília, DF, Brazil; 2 Hospital de Base, Brasília, DF, Brazil; 3 Hospital HVISAO, Recife, PE, Brazil; 4 Clínica COLP, Salvador, BA, Brazil

**Keywords:** Corneal transplantation, Lamellar keratoplasty, Corneal endothelium, Dissection, Tomography, optical coherence, Transplante de córnea, Ceratoplastia lamelar, Endotélio corneano, Dissecção, Tomografia de coerência óptica

## Abstract

**Purpose:**

This study aimed to compare four depths of manual dissection for the preparation of Descemet stripping endothelial keratoplasty lamellae.

**Methods:**

Eye bank corneas were randomized into four groups according to dissection depths: Pachy-100 (incision depth = central corneal thickness-safety margin of 100 µm), Pachy-50 (safety margin of 50 µm), Pachy-0 (no safety margin), and Pachy+50 (incision depth = central corneal thickness + 50 µm). All endothelial lamellae were prepared using a standardized method of manual dissection (Pachy-DSEK). The central, paracentral (3.0-mm zone), and peripheral (6.0-mm zone) lamella thicknesses and incision depths were measured by optical coherence tomography. The 3.0-mm and 6.0-mm zone central-to-peripheral thickness ratios were calculated.

**Results:**

Endothelial perforation occurred only in the Pachy+50 group (n=3, 30%). Central lamella’s thickness in Pachy-100, Pachy-50, Pachy-0, and Pachy+50 groups measured 185 ± 42 µm, 122 ± 29 µm, 114 ± 29 µm, and 58 ± 31 µm, respectively (p<0.001). The overall 3.0- and 6.0-mm C/P ratios were 0.97 ± 0.06 and 0.92 ± 0.14, respectively. Preoperative donor characteristics were not correlated with most thickness outcomes. The planned incision depth correlated significantly with most lamella’s thickness parameters (p<0.001). The overall thickness of the lamella negatively correlated with the planned incision depth (p<0.001, r=-0.580). The best outcome was found in the Pachy-0 group, as 75% of the lamellae measured <130 µm and there was no endothelial perforation.

**Conclusions:**

By using a standardized method of dissection, most manually prepared lamellae presented a planar shape. Setting the incision depth to the central corneal thickness did not result in endothelial perforation and a high percentage of ultrathin lamellae was achieved.

## Abstract (pt)

**Objetivo:**

Comparar quatro profundidades de dissecção manual usadas no preparo de lamelas para transplante endotelial.

**Métodos:**

Córneas humanas de treinamento disponibilizadas foram randomizadas em quatro grupos: Pachy-100 (profundidade de incisão = espessura corneana central - margem de segurança de 100 µm), Pachy-50 (margem de segurança de 50 µm), Pachy-0 (sem margem de segurança) e Pachy+50 (profundidade de incisão = espessura corneana central + 50 µm). Todas as lamelas foram dissecadas através um método padronizado e já publicado (Pachy-DSEK). As espessuras das lamelas (centro, zona de 3,0mm e zona de 6,0mm) foram medidas com tomografia de coerência óptica. A razão de espessura centroperiferia foi calculada aos 3,0 e 6,0 mm de diâmetro.

**Resultados:**

Perfuração endotelial ocorreu apenas no grupo Pachy+50 (n=3, 30%). A espessura central da lamela nos grupos Pachy-100, Pachy-50, Pachy-0 e Pachy+50 foi de 185 ± 42 µm, 122 ± 29 µm, 114 ± 29 µm, e 58 ± 31 µm, respectivamente (p<0,001). As razões C/P aos 3,0 e 6,0 mm foram de 0,97 ± 0,06 e 0,92 ± 0,14, respectivamente. Os parâmetros de características do doador não se correlacionaram com os resultados de espessura de lamela. A profundidade planejada de incisão se correlacionou com a maioria dos parâmetros de espessura de lamela (p<0,001). A espessura de lamela se correlacionou negativamente com a profundidade planejada da incisão (p<0.001, r=-0,580). O melhor resultado foi observado no grupo Pachy-0, em que 75% das lamelas mediram abaixo de 130 µm e não houve perfuração endotelial.

**Conclusão:**

Através de um método padronizado de dissecção, a maioria das lamelas endoteliais apresentou uma configuração planar. O planejamento de profundidade de incisão igual à espessura corneana central resultou em alta porcentagem de lamelas ultrafinas sem ocorrência de perfuração.

## INTRODUCTION

During the last two decades, posterior lamellar keratoplasty has evolved continuously, becoming the first choice for treating endothelial disorders. Descemet membrane endothelial keratoplasty (DMEK) and pre- Descemet endothelial keratoplasty (PDEK) are current modalities that offer near-normal anatomic results through extremely thin grafts, either without or with a little amount of stroma^(1,2)^.

Descemet stripping automated endothelial keratoplasty (DSAEK), which offers an optically good interface and satisfactory visual results, has become very popular, as the automated preparation of grafts is easy and reproducible^(3-5)^. It has evolved to ultrathin DSAEK (UT-DSAEK)^(6)^ and more recently to nanothin DSAEK (NT-DSAEK)^(7)^, providing thinner microkeratome-cut grafts.

Despite such improvement, all these techniques have some important limitations, such as high technical difficulty (DMEK and PDEK) or high costs (DSAEK, UT-DSAEK, and NT-DSAEK).

In such a scenario, we have proposed a new method for manually creating thin endothelial grafts, with low costs, and a smooth learning curve. Recently, we revisited and optimized some steps of the almost forgotten Descemet stripping endothelial keratoplasty (DSEK) procedure.

The Pachy-DSEK method was developed by standardizing and modifying some DSEK steps^(8)^. Some key modifications were as follows: 20 central corneal thickness (CCT) measurements (scanning the central 4.0-mm zone with an ultrasonic pachymeter), incision depth calculated by subtracting a safety margin of 100 µm from the CCT, intraoperative calibration of the diamond knife, and an incision always positioned 5.0 mm away from the corneal center. Consequently, it provided ultrathin grafts in most manually prepared corneas. However, some grafts’ thicknesses were still above 130 µm (20%) or 100 µ (40%)^(8)^.

The primary aim of this in vitro study was to compare the anatomical outcomes of DSEK lamellae, using four depths of manual dissection. The secondary goal was to optimize the safety margin used in the Pachy-DSEK method to create thinner lamellae.

## METHODS

This study followed the tenets of the Declaration of Helsinki and was approved by the ethical committee of the institution involved (registered at www.plataformabrasil.saude.gov.br #39073020.0.0000.5667).

### Donor corneas and randomization

This study included 45 human corneas, stored in Optisol-GS (Bausch & Lomb, USA) at 4°C, consecutively distributed by a public local eye bank (*Banco de Olhos do Distrito Federal*, Brasília, Brazil), and randomized into four groups according to four dissection depths for the manual preparation of the endothelial lamellae:

1. Pachy-100 group: The dissection depth was set by subtracting a 100-µm safety margin from the CCT (defined as the minimum value of 20 central and paracentral measurements).
2. Pachy-50 group: The dissection depth was set by subtracting a 50-µm safety margin from the CCT.
3. Pachy-0 group: The dissection depth was set to be equal to the CCT, meaning no safety margin was considered.
4. Pachy+50 group: The dissection depth was set by adding 50 µm to the CCT.

Randomization and allocation were conducted using Stata v.11 statistical software (StataCorp, College Station, TX, USA). All corneas were assigned to one of the four groups.

Exclusion criteria were as follows: a peripheral scleral rim width <2.0 mm (necessary to properly mount the tissues on the artificial chamber), difficulty in taking the intraoperative pachymetric readings (defined as no reading after three attempts), or CCT >700 µm.

### Stromal dissection

The technique for endothelial lamellae preparation was identical to the previously described Pachy-DSEK technique^(8)^, except for the lack of trephination and a limited 6.0-mm diameter dissection area.

First, we placed the donor cornea in an artificial anterior chamber (Katena, USA) and filled it with Optisol-GS (Bausch & Lomb, USA). A 10-mL syringe was used to adjust the chamber pressure. Then, we removed the epithelium and took 20 ultrasonic pachymetry measurements (Ocuscan, Alcon, USA) of the central and paracentral (4.0-mm zone) donor cornea thicknesses, with the probe angled at 90°. To set the lamellar dissection depth, the minimum pachymetric value was considered and adjusted to the desired final depth according to each group, as described above. An adjustable diamond knife (K3-9500, Katena, USA) was calibrated before each tissue preparation. Then, we performed a 3.0-mm arcuate incision along the 10.0-mm zone. The dissector’s edge was positioned perpendicular to the corneal surface and then gradually moved toward a parallel plane. After the first half of the cornea was dissected, we changed from a straight lamellar dissector to a vaulted one (Katena, USA). Dissection was finished after reaching the 6.0-mm zone and trephination was not conducted. All tissues were dissected by the same surgeon (P.B.). The time between the incision and the end of dissection was recorded.

### Thickness analysis

After lamellar dissection, each cornea was analyzed by anterior segment optical coherence tomography (OCT) (Cirrus HD-OCT 5000, Carl Zeiss Meditec, USA) by the same examiner (CJSJ), who was blinded to both the case number and group.

Then, the OCT images were evaluated by the abovementioned examiner using the Image J software, as previously described^(9)^. In a high-definition horizontal cut, a perpendicular line (using the software’s angle tool) was drawn from the anterior line of the epithelium to the posterior surface of the donor tissue, at the center and 1.5 mm away from the central specular reflection. The peripheral cornea was assessed at the limits of the dissected area (6.0-mm zone) at 3, 6, and 9 o’clock positions (the incision site was considered the 12 o’clock position). The stromal thicknesses above and under the dissection plane were measured at the above-mentioned sites. Finally, each line length was converted from pixels to microns using the software program. Corneal thickness was assessed at the center, 3.0-mm zone, 6.0-mm zone, and incision site.

Regarding lamella thickness, at each corneal zone (center, 3.0-mm zone, and 6.0-mm zone), an average of three readings was recorded. Then, for the overall thickness, an average of these nine readings was recorded. The central-to-peripheral thickness ratio (C/P ratio) was calculated for both 3.0- and 6.0-mm zones. The planned lamella thickness was the central thickness subtracted by the safety margin of each group. The difference between the attempted (planned) and the achieved lamella thicknesses was calculated, considering the central thickness reading of each lamella.

Regarding the incision site, the averages of three readings of the incision depth and of three readings of the full corneal thickness near the incision were recorded.

### Statistical analysis

Statistical analysis was conducted using the software StatPlus for Mac, version v6 (AnalystSoft Inc., Walnut, CA, USA). The normality of data distribution was checked using the Shapiro-Wilk test. Values were reported as mean ± standard deviation and range. Data were analyzed using the Wilcoxon signed-rank test and Spearman’s rank correlation coefficient (rho). Two-by-two and multiple-group comparisons were performed using the Mann-Whitney U test and the Kruskal-Wallis test, respectively. A p-value <0.05 was considered significant.

## RESULTS

### Donor cornea characteristics

The mean CCT was 556 ± 58 µm (range, 465-675 µm). The mean time between tissue preservation and preparation was 23 ± 26 (range, 4-119) days.

No significant difference was found among the groups regarding donor age (p=0.886), CCT (p=0.740), corneal thickness at the incision site (p=0.301), endothelial cell count (p=0.373), and time since preservation (p=0.517). The mean planned incision depths in the Pachy-100, Pachy-50, Pachy-0, and Pachy+50 groups were 468 ± 64 µm, 502 ± 68 µm, 543 ± 54 µm, and 611 ± 53 µm, respectively (p<0.001). The donor cornea parameters of each group are shown in table 1.

**Table 1:** Donor cornea parameters

Parameter	Pachy-100 group (n=12)	Pachy-50 group (n=11)	Pachy-0 group (n=12)	Pachy+50 group (n=10)	p-value^*^
Donor age, years	53 ± 6 (45-61)	46 ± 15 (26-63)	50 ± 18 (25-69)	54 ± 8 (45-64)	0.886
Central cornea thickness^**^, µm	569 ± 63 (484-638)	552 ± 68 (489-665)	543 ± 53 (465-620)	563 ± 54 (480-675)	0.740
Endothelial cell count, cells/mm^2^	2709 ± 392 (2128-2950)	2253 ± 304 (1916-2732)	2254 ± 511 (1538-2976)	2572 ± 909 (1779-3876)	0.373
Preservation-to-preparation time, days	20 ± 11 (8-38)	16 ± 8 (8-31)	19 ± 9 (5-31)	14 ± 10 (4-29)	0.517
Thickness at incision site^**^, µm	710 ± 53 (631-781)	645 ± 77 (510-800)	687 ± 68 (571-788)	644 ± 110 (487-773)	0.301
Planned incision depth, µm	468 ± 64 (380-540)	502 ± 68 (440-615)	543 ± 54 (460-620)	611 ± 53 (530-720)	**<0.001**

All parameters are expressed as mean ± standard deviation (range).

µm = microns; cells/mm^2^ = cells per square millimeter.

Kruskal-Wallis test; bold text indicates a statistically significant difference with a p-value less than 0.05.

** Optical coherence tomography readings.

### Endothelial lamellae preparation

The mean lamella preparation time was 9 ± 4 (range, 4-24) min. No complications were found, except for endothelial perforation, which occurred only in the Pachy+50 group (n=3, 30%).

### Thickness analysis

The achieved incision depth was significantly different between groups (p=0.019). Overall, the shallowest and deepest incisions were measured in the Pachy-100 and Pachy+50 groups, respectively.

**Figure 1:**
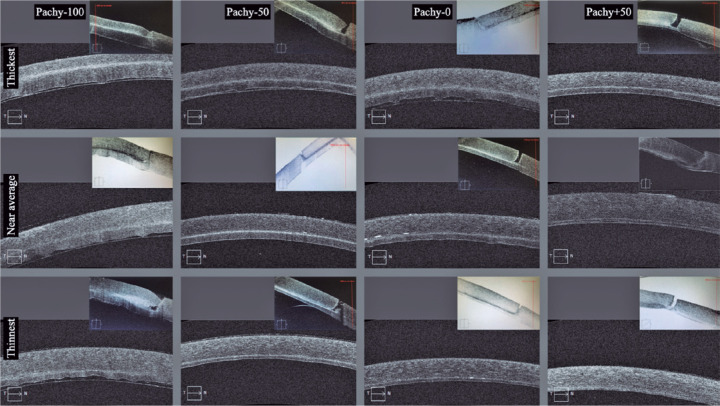
Optical coherence tomography images of the 12 dissected corneas. Within each of the 12 delimited areas, two images of the same case are seen. The inferior larger image represents a high-definition horizontal cut of the center, and the superior smaller image represents a cut of the incision site. From left to right, the four columns represent the Pachy-100, Pachy-50, Pachy-0, and Pachy+50 groups, respectively. The thickest and thinnest lamellae of each group are shown in the top and bottom lines, respectively. The middle line depicts cases whose thicknesses are similar to the thickness average of each group.

Accordingly, the overall thickest and thinnest lamellae were found in the Pachy-100 and Pachy+50 groups, respectively. The overall lamella thickness in the Pachy-100, Pachy-50, Pachy-0, and Pachy+50 groups measured 196 ± 54 µm (range, 123-273 µm), 129 ± 26 µm (range, 72-156 µm), 116 ± 29 µm (range, 64-165 µm) and 62 ± 31 µm (range, 31-107 µm), respectively. The multiple-group comparison revealed a significant difference among groups regarding central (p<0.001), paracentral (p<0.001), and peripheral lamella’s thickness (p<0.001). OCT images of the thickest and thinnest lamellae of each group, as well as the near-average lamellae, are shown in figure 1.

The attempted and achieved lamella thicknesses diffe red in all groups, with an overall deviation from the thickness target of 86 ± 38 µm (range, 12-172 µm). The highest mean deviation was seen in the Pachy-0 group: 114 ± 29 µm (range, 69-164 µm) (p=0.013).

The overall 3.0- and 6.0-mm C/P ratios were 0.97 ± 0.06 and 0.92 ± 0.14, respectively. The 3.0-mm zone C/P ratios were comparable among the groups, except for the Pachy+50 group, which resulted in the lowest values (indicating lamellae with a more lenticular shape).

Most of the thickness-related parameters were significantly different among groups and are summarized in table 2. All two-by-two group comparisons were significant, except for the comparison of the Pachy-0 and Pachy-50 groups (Table 3).

**Table 2:** Endothelial lamella’s OCT-derived parameters

Parameter	Pachy-100 group	Pachy-50 group	Pachy-0 group	Pachy+50 group	p-value^*^
Central thickness, µm	185±42 (118-272)	122±29 (62-149)	114±29 (69-164)	58±31 (24-108)	**<0.001**
Thickness deviation from target, µm	85±42 (18-173)	72±29 (12-99)	114±29 (69-164)	64±31 (31-110)	**0.013**
Thickness at 3.0-mm zone, µm	196±55 (115-274)	124±27 (69-164)	116±30 (64-169)	64±31 (31-110)	**<0.001**
Thickness at 6.0-mm zone, µm	199±53 (137-274)	141±26 (86-175)	119±31 (60-161)	65±33 (34-115)	**<0.001**
Mean overall thickness, µm (center, 3.0 and 6.0-mm zone)	196 ± 54 (123-273)	129 ± 26 (72-156)	116 ± 29 (64-165)	62 ± 31 (31-107)	**<0.001**
Central/peripheral thickness ratio (3.0-mm zone), %	0.99 ± 0.04 (0.94-1.04)	0.98 ± 0.06 (0.90-1.07)	0.99 ± 0.05 (0.90-1.09)	0.90 ± 0.07 (0.77-0.98)	**0.040**
Central/peripheral thickness ratio (6.0-mm zone), %	0.97 ± 0.06 (0.86-1.03)	0.86 ± 0.12 (0.63-1.20)	0.98 ± 0.15 (0.65-1.20)	0.89 ± 0.14 (0.63-1.06)	0.093
Endothelial lamella/total cornea thickness ratio, %	0.31 ± 0.08 (0.19-0.48)	0.22 ± 0.05 (0.13-0.26)	0.20 ± 0.06 (0.14-0.30)	0.10 ± 0.06 (0.04-0.23)	**<0.001**
Incision depth, µm	420 ± 108 (263-572)	434 ± 63 (299-510)	500 ± 67 (411-602)	572 ± 116 (385-704)	**0.019**
Incision depth, %	0.59 ± 0.12 (0.42-0.76)	0.67 ± 0.06 (0.59-0.75)	0.73 ± 0.06 (0.64-0.80)	0.88 ± 0.04 (0.79-0.92)	**<0.001**
Endothelial lamella’s thickness at incision site, µm	289 ± 71 (176-381)	210 ± 43 (140-277)	177 ± 38 (125-232)	73 ± 14 (47-99)	**<0.001**

All parameters are expressed as mean ± standard deviation (range).

OCT= optical coherence tomography; µm= microns; mm= millimeters.

Kruskal-Wallis test; bold text indicates a statistically significant difference with a p-value less than 0.05.

**Table 3:** Comparison of endothelial lamella’s thickness parameters between groups^*^

	Central thickness^†^, µm	3.0-mm zone thickness^†^, µm	6.0-mm zone thickness^†^, µm	Overall thickness^††^, %	Thickness at incision site ^†††^, µm
Pachy-100 vs Pachy-50	**p<0.001**	**p=0.008**	**p=0.023**	**p=0.006**	**p=0.030**
Pachy-100 vs Pachy-0	**p<0.001**	**p=0.004**	**p=0.003**	**p=0.003**	**p=0.006**
Pachy-100 vs Pachy+50	**p<0.001**	**p=0.002**	**p=0.002**	p=0.056	**p=0.002**
Pachy-50 vs Pachy-0	p=0.356	p=0.423	p=0.090	p=0.218	p=0.139
Pachy-50 vs Pachy+50	**p=0.002**	**p=0.001**	**p<0.001**	**p<0.001**	**p<0.001**
Pachy-0 vs Pachy+50	**p=0.003**	**p=0.004**	**p=0.007**	**p=0.004**	**p<0.001**

* Optical coherence tomography (OCT) readings.

† Mean thickness of 3 OCT readings.

†† Mean overall thickness of 9 readings: 3 (center) + 3 (3.0-mm zone) + 3 (6.0-mm zone).

††† Endothelial lamella’s thickness measured just below the incision. µm=microns; %=percentage.

Mann-Whitney’s U test; bold text indicates a statistically significant difference with a *p*-value less than 0.05.

### Statistical correlations

Preoperative donor characteristics were not correlated with most thickness parameters of the lamellae, except for a low positive correlation between donor CCT and achieved incision depth (p=0.015, r=0.407).

The planned incision depth correlated significantly with most thickness parameters of the lamellae (p<0.001), except for the deviation from the target (p=0.294) and C/P ratio (p=0.072).

Only a moderate correlation was found between the planned and achieved incision depths (p<0.001, r=0.574). Moreover, the overall graft thickness negatively correlated with the planned incision depth (p<0.001, r=-0.580).

A high positive correlation was found between the lamella thickness at the incision site and corneal center (p<0.001, r=0.780).

The C/P ratio at the 3.0-mm zone correlated negatively with the achieved incision depth (p=0.035, r=-0.357) and positively with the central graft thickness (p=0.005, r=0.447), although the strength of these correlations was low.

All correlations between donor characteristics and thickness parameters of the lamellae are summarized in table 4.

**Table 4:** Correlations between donor cornea parameters and lamella’s thickness parameters^*^

	Central thickness^†^, µm	3.0-mm zone thickness^†^, µm	6.0-mm zone thickness^†^, µm	Overall thickness^§^, µm	Deviation from target^§§^, µm	Central/peripheral thickness ratio^¶^, %	Achieved incision depth^¶¶^, µm
Donor age, years	p=0.724 r=-0.067	p=0.690 r=-0.075	p=0.412 r=-0.155	p=0.737 r=-0.063	p=0.797 r=0.087	p=0.461 r=0.139	p=0.318 r=-0.195
Time since preservation, min	p=0.149 r=0.260	p=0.115 r=0.283	p=0.266 r=0.202	p=0.146 r=0.262	p=0.054 r=0.348	p=0.697 r=0.071	p=0.635 r=0.088
Endothelial density, cells/mm^2^	p=0.086 r=0.373	p=0.097 r=0.362	p=0.057 r=0.410	p=0.093 r=0.366	p=0.239 r=0.261	p=0.638 r=-0.106	p=0.358 r=-0.216
Donor cornea central thickness, µm	p=0.705 r=-0.059	p=0.186 r=-0.222	p=0.309 r=-0.171	p=0.312 r=-0.170	p=0.247 r=-0.200	p=0.542 r=-0.103	**p=0.015** r=0.407
Planned incision depth, µm	**p<0.001** r=-0.589	**p<0.001** r=-0.605	**p<0.001** r=-0.587	**p<0.001** r=-0.580	p=0.294 r=-0.163	p=0.072 r=-0.298	**p<0.001** r=0.574

Optical coherence tomography readings.

† Mean thickness of 3 OCT readings.

§ Mean overall thickness of 9 readings: 3 (center) + 3 (3.0-mm zone) + 3 (6.0-mm zone).

§§ Final central thickness deviation from the intended thickness.

¶ Considering mean central and 3.0-mm zone thicknesses.

¶¶ Mean of 3 readings of the incision depth.

Spearman’s rank correlation test; bold text indicates a statistically significant difference with a p-value less than 0.05.

## DISCUSSION

Although there is still controversy on whether endothelial graft thickness is related to visual results, there is solid literature to support such. Droutsas et al. found better vision in their sub-100 µm graft group^(10)^. Neff et al. reported a higher percentage of good final vision when grafts were thinner than 131 µm^(11)^. Acar et al. found better vision and higher endothelial cell count in sub-150 µm grafts^(12)^. A multicenter clinical trial reported faster rehabilitation and better visual results in the group of UT-DSAEK grafts (101 µm, range 50-145)^(13)^. Besides, thin and planar grafts might be preferable in triple procedures, as they do not cause much hyperopic shift and interfere less in the intraocular lens calculation^(14,15)^.

Recently, a new method named Pachy-DSEK has shown promising results in generating manually dissected ultrathin lamellae for endothelial transplantation by modifying some graft preparation steps^(8)^. The advantages of DSEK include low cost, smooth learning curve, and technical ease, which make it an interesting option in challenging cases, developing countries, and ophthalmological centers with surgeons in the learning curve. Nevertheless, 20% of the Pachy-DSEK grafts were reported to be thicker than 130 µm.

In this study, despite all the limitations inherent to a manual dissection method, the planned incision depth influences the central, paracentral, and peripheral graft thicknesses. Therefore, the incision depth can be optimized to result in thinner DSEK grafts while still avoiding endothelial perforation and tissue loss.

At both extremes of the dissection depth range, the Pachy-100 and Pachy+50 groups presented the worst results. Overall, the Pachy-100 group resulted in the thickest lamellae, as expected. Likewise, the Pachy+50 group resulted in the thinnest ones (100%, <130 µm; 88%, <100 µm; 50%, <50 µm) but was the only group in which perforation occurred. This indicates that exceeding the CCT when setting the incision depth is more likely to damage the donor endothelium; perhaps, this is the depth limit to be respected in a fully manual technique like this.

At the middle of the dissection depth range, the Pachy-0 and Pachy-50 groups resulted in similar thicknesses. Most thickness readings were lower in the Pachy-0 group, but this difference was not significant, probably because of the small sample size. However, 75% of the grafts in this group were thinner than 130 µm (33% were <100 µm), whereas in the Pachy-50 group, this percentage was only 45%.

In an in vitro study by Tsatsos et al., 10 donor corneas were presoaked in balanced salt solution for 30 min before dissection, and the incision depth was set to the CCT^(16)^. A strong negative correlation was found between graft thickness and donor cornea thickness. Such a correlation was not found in the present study, nor was it in the Pachy-DSEK series, and it might be related to presoaking the donor cornea before dissection. They also reported that 70% of their manually dissected lamellae were thinner than 100 µm. These results compare favorably to ours, except for the ones found in the Pachy+50 group in which 87% of the lamellae measured <100 µm. A larger sample would be necessary to assess whether presoaking consistently results in sub-100 µm grafts.

Only a moderate correlation was found between the planned and achieved incision depths, which suggests that other factors might have had some influence on the incision creation, such as the chamber pressure. Although the overall graft thickness was negatively correlated to the planned incision depth, this correlation was only moderate, which might be explained by slight changes in the depth of the dissection plane when progressing from the incision to the corneal center.

Despite these expected deviations inherent to manual dissection, a high positive correlation was found between the lamella thickness at the incision site and corneal center. This indicates that the peripheral plane of dissection more likely influences the central dissection depth than the incision depth itself.

In the Pachy-DSEK study, the thickness deviation from the target correlated positively with the donor cornea pachymetry^(8)^. Conversely, this correlation was not confirmed by our more recent findings, as this parameter was not associated with any of the studied variables. Larger-sample studies are necessary to confirm this finding.

Besides thickness, another clinically relevant aspect of endothelial lamellae is their shape. DSAEK’s microkeratome-created lamellae tend to have a lenticular shape, explaining most of the hyperopic shifts^(17)^. Conversely, manually dissected lamellae tend to be more planar, an important advantage when cataract surgery is also indicated^(8)^.

By using the OCT-assisted method developed by Yoo et al. to assess the lamella C/P ratio at 3.0-mm zone, we found an overall value of 0.97 ± 0.06 in the present study. This compares favorably to the mean ratio of 0.88 in our in vivo series and to Yoo’s result of 0.85 in DSAEK eyes^(8,14)^. Furthermore, Holz et al. found a relatively faster deturgescence rate at the periphery of endothelial grafts^(18)^. This suggests that C/P ratios tend to increase over time and that our lamellae would probably become even more planar if they were to be implanted in real patients. To the best of our knowledge, this is the highest mean C/P ratio of endothelial lamellae in the literature.

Notably, the study by Tsatsos and the present study report in vitro results. In vitro research in this field has a major bias related to the tissue hydration status, which is substantially time-dependent and expected to change during the period between graft preparation and thickness assessment. Therefore, a higher percentage of ultrathin grafts would be expected after the deturgescence period in real patients.

In their study that included 80 DSEK eyes, Tarnawska and Wylegala measured graft thicknesses in different time points postoperatively and reported an average deturgescence rate of 2.54 µm/day in the first 30 days^(19)^. If this rate were applied to our grafts to predict in vivo thickness, the calculated result after the first month would be 109 ± 42 µm for the Pachy-100 group (50%, <100 µm), 51 ± 22 µm for the Pachy-50 group (100%, <100 µm), and 41 ± 26 µm for the Pachy-0 group (100%, <100 µm). In the Pachy+50 group, most of the calculations would result in negative values.

All the findings of this study suggest that a certain amount of variation in the final graft thickness might be inherent to the nature of manual dissection and less related to the preoperative characteristics of the donor. This limitation is believed acceptable for a cost effective method by which ultrathin endothelial lamellae can be created most of the time.

In conclusion, the thickness of manually prepared DSEK grafts can be improved by setting the incision depth to the CCT, without using any safety margin. The thickness difference between the central cornea (where CCT readings are taken) and the peripheral cornea (where the incision is placed) is probably enough to avoid perforation.

This study is mainly limited by its small sample size. Its strength is to show that a high percentage of ultrathin and planar grafts can be manually prepared by simply optimizing the incision depth. Prospective in vivo studies with larger samples are necessary to confirm these results.
